# Annual Address before the Arkansas Medical Association, at Little Rock, January 6, 1873*We have received through our friend, E. J. Kirkscey, M.D., of Little Rock, Arkansas, a report of the meeting of the Arkansas Medical Association, January 6, 1873—from which we learn that a large proportion of the members were in attendance, and that the Association is composed of the best medical talent in the State. Accompanying the report is the Annual Address of the President of the Association, J. M. Holcombe, M.D., which has been sent to us for publication.—Editors.

**Published:** 1873-02

**Authors:** J. M. Holcombe


ATLANTA

### MEDICAL AND SURGICAL JOURNAL.

VOL. X.—FEBRUARY, 1873.—No. 11.

---

### ORIGINAL COMMUNICATIONS.

---

### ANNUAL ADDRESS*

*Before the Arkansas Medical Association, at Little Rock, January 6, 1873.*

BY J. M. HOLCOMBE, M.D.,

*Gentlemen and Brothers*,—The favor of another year has graciously fallen upon us. Of its bidden pleasures we have tasted, and from its twin cup of sorrow alike we have drunk. The limits within which destiny prescribed we should move bear the visible marks of our presence; and the deeds, which were to be ours in the reckoning, are now legibly written. The same page bears the impress of both our good and evil ones; and whether the balance is to be for or against, until the verdict of eternity is rendered, we can not surely know. "It is done,"—the year itself is dead; and as the mantle of earthly oblivion, save that which history has snatched and garnered, falls upon it, we turn sadly away, as from the grave of buried love; and

* We have received through our friend, E. J. Kirkscey, M.D., of Little Rock, Arkansas, a report of the meeting of the Arkansas Medical Association, January 6, 1873—from which we learn that a large proportion of the members were in attendance, and that the Association is composed of the best medical talent in the State. Accompanying the report is the Annual Address of the President of the Association, J. M. Holcombe, M.D., which has been sent to us for publication.—EDITORS.

yet animated with the hope akin to that which then sustains—that we shall meet it again.

From scenes of human suffering, toil and anxiety, which are peculiarly incident to and color the pathway of our chosen profession, we again, gentlemen, are come together; and here, in this beautiful "City of Roses," we are permitted to clasp anew each brother's hand, and to mutually congratulate and commiserate upon the experience which a twelve-month of actual living has evolved; and not only this, but to cull from this lesson of experience its choicest gems of truth, while more effectually and successfully we can work out the new problem which the opening year presents, and thus swell our contribution to the advancement of our noble art in this active age of progress.

We are not met upon this occasion, the annual reunion of our medical society, to sit in council, as it were, as learned dignitaries, with stiff necks and cool condescension to barely recognize the visible presence of one another. This is the season to unbend, to relax and "rest from our labors." It is the stopping place by the wayside, where, beneath the projecting shades, like the journeying pilgrim on his oasis, we too may drink of refreshing waters, and thus be nerved to renew more ably the conflict which we are waging against disease, the last and greatest earthly foe of man. This is peculiarly the time when we should seek to eliminate the social elements of our nature, and to earnestly cultivate those friendly relations which should ever exist largely among men of the same profession, and laboring unitedly in a common cause. By a close observance of this in our assembling, we shall get upon higher ground, and accomplish that which will inure to and mark the progress of medical history in our young and thriving State.

Of our Association, in general terms now, a word seems fitting. The period of infancy as yet surrounds it. It is barely born and christened, and demands special care and untiring purpose to give it healthy permanent growth and prosperity. This, gentlemen, is peculiarly our work, and by community of effort alone shall we be able to secure for it that prominent recognition in the medical world which was the commendable ideal of its founders. That our number is made up of the foremost men of the profession in our State, is a matter of congratulation and pride.

The character of our Association relates mainly to the advancement of medical science; and we, the living representatives of this body, are its practitioners. With this feature in mind, then, I need not claim your indulgence; nor will I be looked upon as puffed up with self-consciousness, if I devote a few moments to the consideration of our profession—its historical rank and grandeur—the noble spirit inherent in it, as well as the spirit of self-sacrifice which every true physician carries in his bosom. And this I must do, in order to pave the way for the thought I would to-day impress upon and leave with you.

Of the unexpressed or mental estimation in which the people hold the physician, there need be no question. Disease has had claim upon every son and daughter brought forth since the gates of Eden were barred; and no sooner is acceptance rendered, than his services are eagerly sought and tenaciously held, till this claim is either stayed for the time, or its execution consummated. But there is also an expressed appreciation of the physician or man of science, which in the earlier age was especially more marked; and I need not call history to my aid but for a moment upon this point. That rulers and potentates were moved with appreciation, we gather from the fact that they were delighted to honor the fathers of medical science by dedicating their temples in honor of Æsculapius. The genius of Hippocrates, the father of medicine, is extolled by Plato with the warmest enthusiasm; and the rank which Galen held in the medical world has been compared to that which Aristotle possessed in the world of general science. The Greek physician, in coming to Rome, was complimented with the freedom of the Eternal City—a privilege of which that people were extremely jealous and most proudly sparing. By Cicero, the great Roman orator, we were thus made mention of: "Nothing," said he, "brings men nearer the gods than by giving health to their fellow-creatures." The claims of the physician—the real men of science—were appreciated by Napoleon, who conferred upon him high rank and distinction. And in the language of the early Briton, we read from Chaucer the sentiment of his times:

> "In all this world no was there now him like
> To speke of physike and of surgerie."

But, in a word, the foremost nations of the earth, in all ages, have unitedly conferred honors and emoluments upon the scientific physician; and these not through other considerations than as a recognition of his merits and vital worth to man.

The history of medicine, from the earliest dawn until now, has been one continued story of the struggle of its disciples for the relief of suffering humanity, and a continued story of their persecutions, too, by those whose ills they were laboring to ameliorate. But it has been said that "it must ever be to the honor of the medical men of the present age, that though their bread may be said to be gathered through the misfortunes of their fellow-men, they have ever been foremost to point out how these misfortunes are to be avoided—that they have been the first and chief leaders of the sanitary movements everywhere."

But this spirit of self-sacrifice and self-denial, which the daily life of the physician paints upon the moving canvas, will bear meagre mention. Where duty summons, he obeys; there is no hesitation. It matters not whether the misfortune of his fellow-men is instigated by dissipation or brought on by the varied vicissitudes of nature. Its origin he does not stop to question. Is it a calm May morning, when smiling roses adorn the pathway that leads to the luxuriant couch of a suffering nobleman? Is it a mid-winter's night, when freezing snows and piercing winds attend him to the inhospitable hovel of some miserable outcast upon his pestilential pallet, putrid and dying? Or is it the cries of the fallen brave, calling the quiet surgeon mid the rattle of musketry and the booming of cannon, to the very thickest of the fight? It matters not from what source comes the summons, the answer is swiftly wafted back, "I come." Yes, forgetful of self, and oblivious to danger, the noble and philanthropic physician is ever at his post, to minister to the sick, relieve the distressed, staunch the flow of the disrupted life-current, bind up the broken limb, and, when all else fails, to smooth and gild the path with hope that leads through the shadows into the noon-tide brightness of the perfect day. Such, then, are our duties, and such our lives.

We have thus at a glance taken in our profession, noted its high rank in the past, tried to appreciate its intrinsic worth, and to estimate the blessings it confers upon humanity. In view of this, then—and none will say we have drawn upon imagination to paint this picture—in view of our vital importance to men in the aggregate, are we not entitled at the present time to a respectful consideration from the law-controlling powers? Are we not entitled to that protection at least which the law throws around other professions and every-day avocations? And this now is the thought I wish to present—this is the claim I would put forth, "*for legal protection against the impositions from which we suffer*," and for at least that respectful consideration and intelligent regard which is so unsparingly bestowed upon others. But what are these impositions of which we complain, and what their nature, that we should demand or pray that the arm of the law may be interposed to shield us from them? Need I ask this question of you, gentlemen? You, whose reputations have been tarnished, whose faithful and untiring labors have been so much abused, and who have suffered penury and want from this fester upon our profession—this leech, which daily saps from it its life-blood? But the curse which these accursed quacks or empirics entail upon us is but a prelude to the long tale of woe—to the black catalogue of disasters to the human family incident to their life, and unlicensed freedom in the world. Like the locusts in Egypt, they abound and prey upon the vitals of the people like some parasitic growth, eating and destroying the heart's blood of the land. Indeed,

> "With monstrous promise they delude the mind,
> And thrive on all that tortures human kind."

And why should they not thrive and persevere, since they, with their nostrums, receive the implied support of the leading powers of the State, as well as the strong support of the press, and the moral support of the clergy. That they receive the support of the leading powers or law-makers, we gather from the fact that we, who have spent our youth and the vigor of our early manhood, the best days of our lives and our patrimony, in acquiring a scientific knowledge of the various and intricate mechanisms of the human body, and the laws regulating health and disease, are arranged alongside and placed upon an equal footing, indeed, with those swarms of ignorant quacks whose characters we have just painted. We are placed there virtually, since no protection is thrown around us—the real physicians or men of science, in contradistinction from the pretended ones—the ignorant and despicable quacks. We have no legal recognition. The book of laws—the statute of our

State—is silent regarding us, as though our presence even was ignored, and our untiring services of no value to man. But why this fact? Why are we thus isolated, as it were, while all other professions are grouped and hovered under the fostering and protecting wing of the law? For their protection, ere they can command the patronage of the people, it is required or demanded that they be identified and vouched for by properly appointed authority. The minister of the gospel must be weighed as to his qualifications and licensed by his church. The teacher of our common and higher schools must pass critical examination, and be legally authorized before entering upon his duties. And the lawyer, who is to labor to right the wrongs and abuses of society, must likewise be brought to the test, legally licensed, and cause the same to be recorded in the courts of his country ere he will be recognized at the bar, or suffered to practice his profession. Then why, we ask, is not this same safeguard thrown around us, who wear the true badge of scientific practitioners? Is it that health has ceased to be a blessing, or that disease no longer racks with torture and rends the human frame? Or is the traffic in human life of so small moment that it has failed to attract the attention of our law-making powers? Is there no remedy, again we ask? Will unwise legislation, or no legislation at all, longer nourish these impudent mountebanks, who grow fat upon the credulity of the people? Our taxes are imposed and paid that the general good and combined interests of all may be advanced. Would not the favor we to-day are seeking—a like protection and guarantee of our rights, as is accorded to other professions, by the Legislature of our State—take high rank among the so-called "combined interests."

But we have indicated other sources from which quackery and nostrumism — terms which are peculiarly synonymous—draw their encouragement and support—from the press, religious and secular, and from the clergy in person. And this fact, no doubt, has its bearing upon the Legislature in influencing it to take no action regarding the matter. The men who compose that body have serious scruples against combatting the voice of the press; and we need not, indeed, spread out that idea. The fact, however, is to be deplored, that the leading journals of our country, and especially those of religious cast, teem with recommendations to the public of these false pretenders and their like equally false nostrums. And the instances are by no means rare to see them certified to by prominent and well-known members of the clergy; they who, above all others, should labor to protect their flocks from such imposition and deception. The late Professor R. D. Mussey, M. D., of Cincinnati, would not take or allow to come into his house a religious paper that contained a quack advertisement. This example could be commendably imitated, especially by the clergy, and would no doubt be productive of much good. The *Medical and Surgical Reporter*, in speaking of the *New York Independent*, says: "This class of advertisements seems to be the pap on which some of our religious papers live; and they even indicate a willingness to give it the puff editorial." Nor was this all. The editorial columns of this paper had a mawkish, sickly, crawling, crankling sketch and puff of the great mogul himself, who had paid it so much hard cash. Thus this and many of our religious papers are ready to

> "Crook the pregnant hinges of the knee
> That thrift may follow fawning."

And even here in this enlightened and moral city, I have just been pained to see in one of the daily papers the name of one of her distinguished clergy appended to the recommendation of a standing quack advertisement. It is, indeed, astonishing that great and good men—men in high places—should so far forget themselves, and degrade their calling by stooping and pandering to the nefarious schemes of nostrum-vendors and quack doctors. I can not challenge the intent or integrity of these gentlemen of the clergy who have made themselves such willing instruments in the hands of these designing and slippery fellows; yet I insist that they spare from other subjects a small portion of their time to inquire into some of the laws of health and disease—learn something of our profession and its claims—see and answer seriously the questions, Has it accomplished nothing for the good of the human family? Is it that the wonderful discovery of chloroform, which is the greatest boon that God in His providence has given to suffering men, be set at naught? Or is it the "decoy of satan," as expressed by an English clergyman? But is it not a fact, gentlemen, that the brilliant achievements in medicine entitle us to rank by the side, at least, if not in advance, of other natural sciences in point of progress? Have we not fulfilled, and are endeavoring to fulfill, our whole duties as physicians? Are we not canceling every obligation we owe to the communities in which we live? Are the interests of scientific medicine and religion antagonistic? Has not the microscope in the hands of Dr. Beal, a celebrated English physician, overturned the temple of Darwin, in his "primordial form" theory, by demonstrating that vegetable cells have double walls, while those of animals are single? And is it not the proof indeed abundant that the science of medicine in all ages has been a stay and helpmeet to religion, instead of otherwise? Are not their duties and offices, those of the clergyman and physician, largely similar? Then why, we ask, and we are conscious the question cannot be met, are a large portion of the former arrayed against us? Why their influence given to the support of the enemies of legitimate medicine, and alike enemies to the welfare of the people?

We have thus at some length, gentlemen, laid before you the character of the impositions from which we suffer, and, in a word, have shown why they thus flourish in our midst, even though it be to the detriment, not only of our profession, but of the people in the aggregate. They are sustained by the leadin powers in the State; this by non-interference, and directly, by the potent influence of the press, and the high moral tone of a portion of the clergy. This, though it be a shame to us as a people, and a brand upon our civilization, we can not refrain from publishing in your hearing and abroad to the world.

But this, in general terms, we are pleased to call the era of progress and of culture, and boast the advancement we are making in our young and growing State in the application of the useful arts and sciences, and their blessings to our race.

The professional man pretends to stand upon the very pinnacle of science, and looks back upon all time as a gradual field of improvement. Yet we find that we are more than two hundred years behind the great commonwealth of Massachusetts in legal enactments for the protection of the public, as well as the profession itself, against empiricism and quackery, and more than a century behind the Empire State of New York. Indiana has recently adopted stringent laws regulating the medical practice in her borders, which reflects honor upon her lawmakers. The great young State of Minnesota has thrown a safeguard around her citizens, and protected the rights of legitimate medicine, by enacting stringent laws in March, 1869. They are indeed worthy of emulation, and speak well for the enlarged views of her wise legislators.

The first section provides that it shall be unlawful for any person to practice or prescribe medicine who can not produce a certificate of qualification from some county, district or State medical society, or who has attended at least two full courses of medical lectures, and is of good moral character.

The second section provides for fine and imprisonmeut for violation of section first, while the third section provides for the registration of the certificate or diploma in the office of the district clerk. Nor are the above States the only ones which have guarded in this manner the highest interests of their people. Nearly the entire Northwest has taken similar action, the fruits of which are there most pleasingly apparent. But I will not instance others.

I have worried your patience, perhaps, gentlemen, and have been over-tedious upon this subject, but I deem it a most vital one to the whole people, and one which cannot be touched lightly upon. My desire and aim has been to elicit some public expression from this body, trusting that in so doing

> "Strong truth may thus the public mind persuade,
> And spoil the fruits of this nefarious trade."

Yes, to speed the time when the real or genuine physician of our State shall be relieved from the odium and disgrace which attaches to the name of "Arkansaw Doctor," an epithet growing out of unbridled and unrestrained quackery, which is a stench in the nostrils of all decent medical gentlemen, both here and the wide world over.

Now, it cannot be denied that here in our State, in every community, the most ignorant and brainless mountebank can hang out the badge of our profession and pretend to practice medicine, and collect his bills before the courts of the State, with as many rights at law as we, who have spent our fortunes and the best of our lives in acquiring a certain degree of mastery of the medical science. And why is this in still closer sense than before indicated? Because the public or people, like these deceivers, are themselves ignorant. They are not sufficiently informed upon the simplest physiological and pathological laws, causes and prevention of disease; or, in other words, they know comparatively nothing of the workings of that wonderful and beautiful casket which God created after his own image for their temporary dwelling place. And other conclusion than this we can not deduce from our intimate knowledge of human nature. The instinct of self-preservation is apparently one of the first implanted in man. If his life be in peril, or aught menaces his personal weal or safety, every faculty of his nature is aroused, and every latent effort put forth, to free himself from the danger surrounding him. Could he appreciate, as can we, who, from a knowledge of science, can look behind the curtain, that danger attends his every footstep, while the medical practice is thus defaced by no fixed line of demarkation between the real and pretended practitioner, we can fancy his prompt action, and the aid he would prayerfully invoke, to help inaugurate an era of safety to human life. And such an era, indeed, is now demanded in our State. It can be had at the hands of our Legislature, and we humbly trust that during the present session it will imatate the wise examples from abroad which we have noted, which is so palpably in sympathy with the highest good of all classes.

But, leaving this subject now, with the hope that what we have said is not in vain, we come to another, which we cannot refrain from briefly noticing at this time; and that is the isolation of our profession, or, in other words, our unrecognized claims to public preferment and places of trust.

Is it not remarkable, gentlemen, that a profession like ours, whose every effort is directed to the well-being of the public—"the preservation of health, the cure of disease, and the physical perfection of man"—should be so neglected and ignored by the State? How rarely do we meet the man of science in the legislative halls, or in other important positions of honor and trust? And the exclusion has been so universal that it almost seems out of place to have the name even of the physician mentioned in connection with the affairs of State. Mark the difference now between this and European countries. The medical profession is represented everywhere. Directly the opposite of what we complain is there apparent. The presence of the physician is there courted by all in high circles—at the court of the sovereign, among the peerage, and in the legislative bodies—and his opinion touching the interests of the profession and the public generally, is received with deference and respect. That this is not the case here, we opine that the people, in part, rest upon the hackneyed phrase that "science is its own reward," and hence consider our public preferment uncalled for and out of place. Of all the other professions I need not remark that they are rewarded by the State and people for distinguished services, while we must look alone to that proud consciousness for our reward in having discharged fully our duties to our fellow-men, or to the pecuniary benefits accruing from our hard labor; and this latter thought, granting that the consciousness of duty done is a part of our reward here, what do we find from a consideration of our pecuniary benefits. And this, too, is a matter of vital importance to the physician of our State—timely pecuniary remuneration for our services. Now, when we consider the arduous duties, hardships and anxieties incident to our profession, and then contrast its remuneration in this sense with that of other professions and avocations, we can not fail to observe the injustice done us. How often do we meet the physician borne down with the infirmities of old age, bleached with the frosts of many winters of hard service for suffering humanity, in almost abject poverty, and not often this in other professions. With the same prudence and attention to their calling, it is seldom they do not gather enough of the fruits of earth to smooth the path and solace the decline of life. But that we are the exception, gentlemen, I do not propose to blame others so much as our own individual selves. The fault is ours in making no effort to incite or educate the public mind to a prompt payment of our fees.

We have not, as we should have done, also, with the people, called the attention of the law-makers to our wants and necessities in this direction. If we are indifferent and careless of our own interests, and silent regarding them, how can we expect others will espouse our cause and advocate it for us. But consider for a moment, if you please, the popular mind regarding our claims financial. The physician's bill, to the majority of the people, is regarded as an outside matter—one that can be settled at any time—and is not be reckoned as a valid claim in the general summing up. It is rather looked upon as a *charity*

to be bestowed upon the "good doctor who has been so kind to us," but even this not until the grocer, the tailor, and the *liquor dealer* have all been satisfied, together with every other "debt of honor." And there is still another class not so free-hearted as the aforesaid—those who are never guilty of paying a dollar to a physician, and yet at the same time constantly receiving his services. When human nature finally revolts, or the pressing needs of the physician impel him to call for a small portion of his fees, he invariably is abused for his temerity, and insultingly told his services are no longer required; while the enraged patron immediately sends for his competitor, who, responding, in time meets with the same rebuff. So they go the whole round of the trusting faculty, and never pay a fee. Among other professions—and we will not dwell upon this—there is either the protection of the law against such impositions and outrages, or they are so circumstanced as to protect themselves. The lawyer secures his fees before he enters upon the case of his client; the merchant, ere he will make advances, must have a mortgage upon both personal and real estate; the common laborer is secured for his work by the operation of law upon the growing crop; the mechanic's lien attaches when his work is complete; while the trickery of the law, in its exemption clauses, shields the non-paying public, and thereby defrauds the physician of his hard earnings—for there is certainly not more than ten per cent. of the whole population who have above seven thousand dollars, which amount, in our State, is exempt from execution. Here, again, as elsewhere instanced, we as a profession seem to stand alone—to be isolated from the rest of toiling humanity.

But I hasten to conclude. The thoughts I have to-day presented for your consideration are those which I have long felt should take possession of us, and be urged by us, singly and collectively, upon the people at large. Unless they are—unless we as a profession gird up our loins and labor unitedly to correct the abuses from which we suffer, and invoke and receive the aid and sympathy of the controlling powers, our lives here will, in a large measure, be spent in vain, and our names go down and rot beside the vile pretenders who now walk beside us. I may be considered harsh in my denunciations of the "quack fraternity." I would to heaven I could say more—sufficient, indeed, to awaken the people from their lethargy regarding them, and incite the law-makers of our State to pass such laws as will protect the people from them in every shape and form they may assume. This done, and the other abuses, which are in part the outgrowth of this, would soon be known no more. Let us hope that another year, when we shall again assemble as to-day, we may congratulate each other that something has been accomplished for us, and the people as well, in this direction.

And now, in closing, gentlemen, in a word, allow me to tender my sincere thanks for the high compliment your voluntary suffrage has conferred upon me by electing me to preside over your deliberations. That the mantle of my distinguished predecessor could have fallen upon the shoulders of one more worthy, I must say in all candor I had wished. But you saw fit to honor me, which favor from you I shall never forget. You will allow me, in sympathy with the thoughts I have presented, to exhort you to guard this, our young Association, for it is the only safeguard thrown around the genuine practitioners of our profession. Promote its interests in every honorable way—for by giving to it new life and material worth, we not only contribute our share to the advancement of science, but in so doing entail blessings upon our fellow-men. I thank you, gentlemen, for your kind attention and unwearied patience, and now bid you God-speed in your noble endeavors to bless and elevate mankind, and to soften the asperities and ease the pains incident to human life; and I would that each of you, in the beautiful language of Bryant, may so order your lives—indeed,

> "So live, that when thy summons comes to join
> The innumerable caravan that moves to the pale realms of shade,
> Where each shall take his chamber in the silent halls of death,
> Thou go not, like the quarry slave at night, scourged to his dungeon;
> But, sustained and soothed by an unfaltering trust,
> Approach thy grave like one who wraps the drapery of his couch about him,
> And lies down to pleasant dreams."

